# Ageing, the digital and everyday life during and since the Covid-19 pandemic

**DOI:** 10.3389/fpsyg.2023.1168340

**Published:** 2023-09-27

**Authors:** Wendy Martin, George Collett, Chris Bell, Amy Prescott

**Affiliations:** ^1^College of Health, Medicine and Life Sciences, Brunel University London, Uxbridge, United Kingdom; ^2^William Harvey Research Institute, Queen Mary University of London, London, United Kingdom

**Keywords:** ageing, digital, time, space, rhythms, everyday life, COVID-19 pandemic, social connectivity

## Abstract

**Introduction:**

During and since the Covid-19 pandemic there has been an intensified integration of digital technologies into the everyday lives of older people. We do, however, know little about the ways in which older people incorporate digital technologies and communications into their daily lives and their own meanings, embodiment and experiences of the digital during and since the Covid-19 pandemic.

**Method:**

The aim of our research was to explore the use of digital devices during and since the Covid-19 pandemic and to identify facilitators and barriers to incorporating digital devices into everyday life. The research involved a series of online focus groups with people aged between 63 and 86 years living in the United Kingdom and were conducted in 2022. Each focus group lasted around 90 min and data was audio-recorded and transcribed. The data was analysed thematically.

**Results:**

From the analysis, three interconnecting whilst analytically distinct themes around the meaning and experiences of using digital devices in everyday life during and since the pandemic, are thematically presented as: (1) Incorporating the digital into everyday life; (2) Social and digital connectivity; and (3) Challenges and limitations of the digital in everyday life.

**Discussion:**

The research has provided insights into the way digital devices were used by older people during and since the Covid-19 pandemic. In particular, we highlight the increasing importance of digital connectivity and the ways in which older people actively engage (and resist) technologies of communication in their daily lives; and the significance of embodied co-presence and the immediacy of shared space and/or time is highlighted.

## 1. Introduction

### 1.1. Ageing and the Covid-19 pandemic

The Covid-19 pandemic significantly impacted the everyday lives of older people. Firstly, the virus itself caused a disproportionately higher number of excess deaths in older adults (Rossen et al., 2020) which subsequently led to heightened fears and anxieties associated with this demographic (Agrawal et al., 2021). This elevated level of concern for the wellbeing of older people also led to an increased emphasis on the possible vulnerabilities of older people in terms of rules and regulations around social engagement and social isolation.

One of the consequences of this can be seen as a heightened sense of being “old” and an enhanced sense of vulnerability among some older people, more so than before the pandemic (Shrira et al., 2020). This was demonstrated by a study of letters written by older adults in Finland during the pandemic (Leinonen, 2022). Leinonen presents three key themes that were described as: (1) *not being*: that denoted social isolation and changes of identity, particularly the idea that one is old and frail and with needs; (2) *not having:* that describes the invisible virus, not seeing or hearing people, items missing from shops; and (3) *not doing:* the withdrawal and disengagement from other aspects of life but also new, different freedoms that has been described as the “unlived life” and appears to represent a shared experience of older people during the pandemic.

These issues amongst older people were moreover exacerbated by rising ageism that appeared to emerge during the pandemic (Fraser et al., 2020). This appeared in a number of forms, with the most prevalent being the narrative that all older adults were significantly viewed as at high risk and perceived as more vulnerable than the majority of the rest of the population. Another area that became more salient during the pandemic were predominant ageist narratives around older people and the use of digital technologies in everyday life (Ehni and Wahl, 2020; Mariano et al., 2020; Swift and Chasteen, 2021).

### 1.2. Ageing and the digital

Digital technology is a realm in which older adults have consistently experienced ageism in a number of ways. Firstly, the perception of older people as being incompetent and unwilling when it comes to digital technology serves as not only a prejudice, but also a barrier towards greater use of this technology in this population (Gates and Wilson-Menzfeld, 2022). The increasing integration of the digital into everyday life (something exacerbated by the Covid-19 pandemic) also may leave older people who are not fully assimilated into digital use, at a clear disadvantage in a number of ways (McDonough, 2016), further exacerbating this digital ageism. In addition to these issues, the vast majority of digital technologies and platforms are simply often not designed with older adults in mind, adding another layer of discrimination and ageism into the use of digital technology (Rosales and Fernández-Ardèvol, 2020).

Despite this, digital technology may provide a wealth of benefits to older adults including memory aids (Atkinson and Barker, 2020), mental health and cognition (Yoo et al., 2022), facilitating communication and social connections (Marston et al., 2019) and a range of wider health and wellbeing benefits (Augner, 2022). There is also a wealth of evidence showing that many older people are embracing digital technology and in particular usage of mobile phones, emails and the internet are common in older people in developed countries (Marston et al., 2019). Older adults are a heterogenous group with technology usage varying widely amongst them and whilst rates of digital participation decline with age, there is still a high degree of variability at all ages up to the oldest old (Taipale et al., 2021). In addition to this, older people are often taking a proactive role in not just using digital technology but also in helping to develop and create digital technologies, particularly in fields where they have been identified as being particularly useful for this demographic (Kania-Lundholm and Manchester, 2022). There are signs that the producers of digital technology and platforms are starting to understand the importance of older adults as a heterogenous and growing section of the population be included in the design of technologies in order to maximize their function (Mannheim et al., 2019; Peine et al., 2021). This could prove to be very important moving forward, as digital technology provides a huge range of potential benefits for older adults including physical functioning, information access, facilitating social connections and health monitoring (Sheng et al., 2022).

### 1.3. Older adults, the digital and Covid-19

The gap between those who have access to digital technology and those who do not has been coined the “digital divide” (Van Dijk, 2006), and extends to a number of demographics including those without access in developing countries, rural communities, those with certain disabilities and older adults (Van Dijk, 2020). Access to digital technology provides only the first level to this divide, with knowledge and skills for usage and the ability to use digital devices to achieve specific ends representing second and third levels to the divide, which has become more pronounced as a result of the Covid-19 pandemic (Aissaoui, 2021). It has been suggested that this divide exists as a result of these practical difficulties rather than an unwillingness amongst certain groups (including older adults and rural dwelling groups) to adopt such technology (Freeman et al., 2022). Digital technology usage has increased in, and heavily influenced a number of key areas of society including healthcare, education, workplaces and home life and extend to a range of both hardware and software versions (Vargo et al., 2021). This innovation and subsequent reliance on digital technology in a number of areas of life has only exacerbated many of the inequalities that already existed between groups with greater access to and familarisation with the digital and those without (Lai and Widmar, 2021). Whilst tired stereotypes of older people as uninterested or unskilled users of digital technologies have waned, concerns over a digital divide remain and necessitates more about the meanings and experiences of ageing and the digital in everyday life (Peine et al., 2021).

### 1.4. Ageing, the digital and everyday live during Covid-19 pandemic

Contemporary and global societies are characterized by changes in meanings and experiences of space and time. There has been a move from predominately face-to-face relationships in which time and space are inextricably linked, to an increasing separation of time and space resulting in more disembedded and distanced social relationships (Giddens, 1991). Massey (1994) criticizes the dualist tendency of conceptualizing space and time as bounded and separate, and instead states that space and time are intimately interconnected, and are constructed out of social relations, within a context in which social relations are dynamic and changing. Interconnections of time and space are seen to coexist in everyday life and shape the meanings, flows and experiences of daily routines and practices (Lager et al., 2016; Lyon, 2019). The concept of rhythms in everyday life draws on Lefebvre’s Rhythmanalysis (2004) and argues that time and space are inextricably interwoven: ‘everywhere where there is interaction between a place, a time and an expenditure of energy, there is rhythm’ (Lefebvre, 2004, p. 15). The ordering of everyday life and the flows and rhythms are moreover derived from daily practices (Lager et al., 2016). The emergence of cultural gerontology has further highlighted the significance of meaning and lived experiences of people in later life in the context of time, space and everyday life (Twigg and Martin, 2015a,b).

A focus on everyday life brings attention to the taken-for-granted, the ordinary, the mundane, the day-to-day, the habitual, and the rhythms, and routines of daily life (Katz, 2018). The Covid-19 pandemic disrupted the daily lives of older people and it is in this context that social connections, routines and rhythms of everyday life significantly changed alongside the ways that older people increasingly used digital devices and technologies. During the Covid-19 pandemic in the United Kingdom there were significant impacts on daily lives for at least 2 years from March 2020 with a series of public health restrictions including lockdowns and social distancing, when mobilities and movements were very limited and people were at times mainly required to stay at home. The rhythms and routines of everyday life were disrupted and the usual connections with friends and family and wider contexts of work, care, and leisure in-person were limited.

Digital devices, information technologies and mediated systems of communication have increasingly shaped the social worlds of people as they grow older (Peine et al., 2021). Digital technologies permeate everyday life and have become interwoven with our identities, narratives, social relationships and the rhythms and routines in everyday life. We do, however, know little about the ways in which people in mid-to-later life incorporated digital technologies and communications into their daily lives and their own meanings, embodiment and experiences of the digital during and since the Covid-19 pandemic. The aim of this paper is to highlight some of the ways that older people performed, mediated and experienced the use of digital technologies during and since the Covid-19 pandemic; to explore the meanings and experiences of digital technologies in everyday life; and to identify facilitators and barriers to incorporating digital devices into everyday life.

## 2. Data collection methods

The research involved three online focus groups with people aged between 63 and 86 years living in the United Kingdom and were conducted in 2022. The study was approved by the Brunel University Research Ethics Committee (Reference: 30547-LR-Dec/2021-36960-1). Informed online consent was gained from all participants prior to data collection.

### 2.1. Participants

This study involved members of the Brunel Older People’s Reference Group (BORG) which is a database of approximately 262 local adults aged over 50 years old who are interested in participating in research studies and have agreed to be contacted. BORG members live in West London and the surrounding area. Those on the BORG database were sent a general email inviting them to express an interest in the research. The study was also advertised at BORG community research online events and advertised during monthly online BORG meetings. Those who expressed interest were sent study information and consent forms and were also asked to share the study information with other potentially eligible participants. In addition to being aged 60 and over, participants were eligible for the study if they currently use at least one digital device or technology, such as a tablet, laptop, smart phone, or wearable wristbands (e.g., Fitbits, digital watches, and others) in their everyday life.

A total of 12 participants aged between 63 and 86 participated in the study (mean age = 76 years). Most participants were female (*n* = 10), and all identified as White ethnicity and were homeowners (Table 1; participant names replaced by pseudonyms).

**Table 1:** Participant demographic information.

Pseudonym	Focus group	Age	Gender identity	Marital status	Ethnicity	Employment status	Previous/current employment	Home-owner
Gloria	1	82	Female	Married	White	Retired	Teacher	Yes
Margaret	1	86	Female	Single	White	Part-time	Nursing	Yes
Katherine	1	74	Female	Divorced	White	Part-time	Secretarial	Yes
Roy	1	78	Male	Married	White	Part-time	Information technology/Rowing coach	Yes
Corinne	1	75	Female	Married	White	Part-time	Priest	Yes
Yvonne	2	72	Female	Married	White	Retired	Systems analyst	Yes
Shelly	2	78	Female	Married	White	Retired	University staff	Yes
Bill	2	74	Male	Married	White	Retired	Healthcare project manager	Yes
Janet	2	72	Female	Married	White	Retired	Organisational development	Yes
Sylvia	3	79	Female	Divorced	White	Retired	Nursing	Yes
Irene	3	79	Female	Widowed	White	Retired	Teacher	Yes
Pauline	3	63	Female	Married	White	Part-time	Teaching assistant	Yes

### 2.2. Data collection

Due to the ongoing pandemic and possible increased vulnerability to adverse health outcomes of Covid-19 amongst older adults, and the enhanced Covid-19 restrictions of the ethics committee that limited in-person research, the data was collected using a series of online focus groups conducted using Zoom software (version 5.9.3). The focus groups were led by two researchers—one as a facilitator and one as a moderator and took place between 25th February 2022 and 28th March 2022.

Focus groups are moderator-facilitated group discussions organized to explore a specific set of issues and are distinguished from group interviews in that there is a component of ‘group interaction’ which contributes to the research data (Kitzinger, 1994). This methodology was chosen because focus groups are an efficient way of gathering multiple perspectives and opinions on the use of digital devices during Covid-19 pandemic. It is also an effective means of eliciting meanings, insights and norms and values among social groups (Barbour, 2014).

There were five participants in focus group 1, four participants in focus group 2, and three participants in focus group 3 (*N* = 12). The focus groups were semi-structured meaning that each focus group followed an interview schedule but with the opportunity for free discussion if the discussions (for example, in relation to embodiment, surveillance, and data tracking) were relevant to the research question.

The focus groups began with a preface to reintroduce the study, the researchers, to explain the purpose of the focus group, and reiterating rules of confidentiality and voluntary participation. After introductions, discussion was elicited by asking each participant “are digital technologies important in your everyday life since Covid-19, and why?” and “has your use of digital technologies changed in Covid-19? If so, in what ways?”

Discussion was also elicited by asking participants what digital technologies they had used since Covid-19 pandemic but not before the pandemic, what do they enjoy about digital technologies, and what worries or concerns do they have about digital technologies. We also explored the use of digital devices in specific contexts, namely the use of digital devices in healthcare, the use of digital devices as memory aids (e.g., daily reminders, organizers), and the use of digital devices to enhance and maintain social relationships.

Due to more exposure and an increased use of online means of communication during Covid-19, many of the participants were used to engaging with online means of communication. Not all of the participants were used to Zoom so that we took time to get started and allowed participants to get used to the technology. There was one participant who was unable to join online and was supported by one of the researchers by telephone. So, whilst online focus groups can be an effective and efficient means of eliciting data among older people, it is possible that our focus groups did not include potential participants who may not feel comfortable with the group conversations online, and/or do not have access to the digital means.

Group interactions are not only important to elicit insightful data but observations of the interactions are data for analysis (Barbour, 2014). The focus groups were facilitated to enhance conversations between the participants as well as with the facilitator. There was attention given to the sensitivity of the topic as the Covid-19 pandemic had been a complex and difficult time for many people. The participants on the whole appeared to appreciate and gain from the opportunity to share their experiences and insights around the Covid-19 pandemic.

### 2.3. Analysis

Each focus group was audio recorded, anonymized and transcribed verbatim. The transcripts were then analyzed using thematic analysis (Braun and Clarke, 2006, 2022). Firstly, each focus group was listened back, and the transcripts were read through (accompanied with moderator notes from each focus group) to familiarize with the data and consider potential codes. Preliminary codes were made for each transcript with the research questions in consideration (Researchers GC and CB). The preliminary codes were then refined into a coding table to facilitate identification of themes (GC and CB). Themes were generated based on the salience and relevance to the research question. Each theme and the codes comprising the themes were reviewed by all authors before finalizing a set of themes which best describe the data.

Whilst there are debates about “saturation”, a systematic review argued that saturation can be reached with small sample sizes (Hennink and Kaiser, 2022). Following the series of focus groups many of the key themes were evident across the focus groups and there was richness from the online group focused conversations and interactions. This paper can be described as not a total account of the experiences and perceptions of older people about their digital devices but has captured data from a diverse range of older people, at a certain time and in a particular place. The findings thereby provide important insights into the experiences and perceptions of ageing and the digital during and since the Covid-19 pandemic.

## 3. Results

Three interconnecting whilst analytically distinct themes around the meaning and experiences of using digital devices in everyday life during and since the pandemic, are thematically presented as: (1) Incorporating the digital into everyday life; (2) Social and digital connectivity; and (3) Challenges and limitations of the digital in everyday life. The themes were generated by an interrogation of the data that was informed by both the key research questions and the narratives generated amongst the participants.

### 3.1. Incorporating the digital into everyday life

At the start of the pandemic, the changes to the everyday routines and rhythms of the participants were significant. The participants described their vivid recollections of this time, it felt momentous within their lives. The public health messages and regulations limited movements, and were especially focused on older people, with several of the participants or their partners needing to shield, in which there was guidance to socially distance and to stay at home. Many of the participants described this time with a sense “fear” and continual feelings of worry:

there is always at the back of … especially at the beginning, was the fear (Gloria).

The taken-for-granted aspects of everyday had been challenged and the everydayness of routines and rhythms were questioned and became noticeable (Leder, 1990; Turner, 2004). The significance of everyday routines enables people to manage their sense of vulnerability, described by Giddens as “ontological security” (Giddens, 1991). Daily routines and norms can often be disrupted in the context of physical and social risks as people grow older, in this case, the pandemic represented a risk in which a sense of “ontological security” needed to be renegotiated (*cf.* Turner, 2004). The integrity and logic of the ageing body and daily routines were questioned and the taken-for-granted nature of embodiment and the rhythms of everyday life increasingly challenged. As the habitual and routinised rhythms of everyday life were disrupted, the participants were actively looking to develop new flows and rhythms in their daily lives:

There’s something about concentration, concentrate … being more aware of things … and being a bit more disciplined as well in a way, I think as an older person and a person … I’m … largely not regulated by going to work anymore and that sort of thing. So I think these … that having to look after ourselves better and being shut down and … throughout Covid, we had to be inventive and … and think about what we might do. (Corinne)

The extent to which the participants were influenced by their experiences of lockdown and social distancing was different. In particular, some participants needed to leave the home daily to engage in paid work. For example, one participant was a teaching assistant, and they were often going into the workplace, although the means of teaching during a pandemic required new digital skills:

so I had to learn a lot then. Google Classrooms, I never want to see that again. (Pauline)

The pandemic not only resulted in a heightened sense of vulnerability but the everyday movements and mobilities of the participants were limited. Most of the participants were no longer able to freely meet others in-person outside their household. This is when the participants started to increase their use of digital technologies:

But you are right, on the 22nd of March 2020, did I have any idea what something called Zoom or Teams were?! Didn’t have a clue. (Sylvia)

Most of the participants increased their use of digital technologies, for some at first with a sense of reluctance until the realization that the pandemic was long term, and the purpose was to predominately maintain social connections and social activities. This included connections with family and friends, with the participants starting to change the ways they used digital technologies:

But with … as regards communicating, we used to use Facetime all the time but then we wanted to start sharing things …. So my daughter set up a Zoom account which her children could use, so they could Zoom me and share things with me (laughing) on their screen, and so we got really into sharing screens and things. (Irene).

The participants used a range of devices including mobile / smart phones, laptops and computers and iPads. There was also a wide range and increasing use of a variety of means of communication and digital technologies that included social media, WhatsApp, Teams and Zoom to enhance sharing and communication with friends and family.

The participants also showed how they maintained contact with social groups and social activities. This included social connections and activities associated with the church, music and choirs, hobbies, learning activities, dieting, dance and exercise and also participating in paid work and volunteering:

I was having some private … some French lessons outside U3A and the teacher adopted Zoom quite quickly. My Pilates class went on Zoom. I used Zoom socially with friends to keep up. (Janet)

As many events went online, some participants took the opportunity to engage with the arts that they may not have physically travelled to and therefore some new opportunities opened up:

But digitally Edinburgh was fantastic because they did the Book Festival on-line, so we were able to listen to people who had probably never got … went up there, probably would not have even gone to see and discovered new authors and that, so that was good. (Gloria)

Participants talked about the importance of being together and passing shared time online whilst being in different spaces. They were often sharing and doing activities, such as playing cards, drinking wine or doing crafts. At times this eased the sense of isolation and the embodied doing and performing of an activity meant that conversations did not need to be continual but intermittent and from time to time as the participants shared an activity:

And one of my friends, during the depths of lockdown, when we were getting a little bit challenged for things to do, we were doing sort of craft afternoons on Zoom. (Janet)

Other online activities were mainly done alone, such as online shopping, browsing online, playing games and reading. Some activities that were moved online were seen as useful and engaging, whilst other activities, such as, online jigsaws, and most activities around the choir and orchestra, were experienced as more problematic:

Yeah, so we were all just basically singing to ourselves. We could hear him and what he was playing, but we could not hear each other, unless he said ‘unmute’ and then we could all talk to each other. But while we were singing it was very weird. (Pauline)

Adapting to social activities and connections online did take some time. The momentum online was described as different to face-to-face activities, as there was the need to adapt to using the technologies that included logging on, turning on videos, using the mute buttons, and way that online communications often work more effectively when each person takes a turns to speak. In particular, the participants missed more intimate connections when in small groups or the momentum of conversations can occur in physical space, but is more limited in digital space:

And that’s really weird when you have got hundreds of people coming in and … you cannot sort of sit and chat to people like you can in a small group! (Pauline)

The experience and meaning of space were important, as many of these connections took place at a distance, in different spaces, but at the same time. The participants did at times compare how the difference in space from a shared physical building was to the experience online and the ways the activities changed and developed:

I enjoyed all the work that I did during Covid, which was very different from being in a church building, to doing it on Zoom. (Corinne)

Boundaries around space can be drawn and re-drawn, for as Massey (1994) argues space does not have ‘fixed’ meaning, but instead meanings can be made and re-made in the spaces and moments the practices take place, as the digital, material and social relations intertwine and interconnect. Meaning around space can thereby change depending on context, as Goffman (1959) showed how our presentation of the embodied self can be performed differently depending on whether we are in spatial contexts considered as front or back spaces. In areas that are considered private (back stage), people often feel more relaxed and less concerned about their embodiment, whilst to engage with others can necessitate a more formal and public presentation (front stage) (*cf.* Peace, 2022). The visual nature of online connections blurs the boundaries between more private (back stage) and the more public (front stage) when presenting the embodied self:

I mean at one time I used to be frightfully worried about you know how do you look and what … you know have I … should I put lipstick on or whatever? Now I … you know you … it’s take me as you find me and that’s it, so … I’m more relaxed about it now. (Katherine)

This was sometimes expressed as a more informal way to connect when within the more private space of the home:

in a way it was quite handy because you did not have to get dressed up or … (laughing) … get anywhere or get so organized, you could just sort of like finish your dinner and just come into the next room, turn on … (Pauline)

There were differing views about moving from in-person to online deliveries for food shopping during the pandemic. As shopping for food was designated as an essential activity, there was the possibility of going to the supermarket in person, whilst socially distanced, or ordering online:

At the beginning they offered us you know could they do anything, give us any food that we wanted or do any shopping. But … like other people have said, we had neighbours who would do it and I did carry on doing the shopping, very carefully, because I could. And I wasn’t very good at doing shopping on-line. We live within sort of Sainsburys, Tesco’s and Lidl’s, within sort of four, five minutes, so I wasn’t a very good bulk shopper because I knew I could just pop down and get something. (Gloria)

The decisions around shopping in person or online delivery involved their own sense of risk, the practicalities and everyday routines around shopping and social connections within the locality, such as, neighbours:

because of lockdown, all our shopping is done on-line and she looks around at Tesco and Waitrose and etc., to see where the best buys are. The other thing is we … our neighbours on both sides were not shielding as much as us, and they went shopping for us, so we got to know our neighbours even better than we knew them, they are lovely neighbours anyway, but we got … I got to know them even better. (Roy)

At the time of the interviews, the United Kingdom had experienced three lockdowns, and there were the beginnings of the public health restrictions around social distancing being reduced, alongside a vaccination programme. The participants had started to meet in person for some social activities. At the same time, after the long period of time conducting activities online, the participants highlighted some of the perceived benefits of continuing with some digital connections, that included, being more efficient with time, fitting the activity around other routines, not needing to travel, and not having to go out in wet weather. Many participants described the possibility of social activities being more hybrid, in which people could choose to be online or in person, and this was considered in some contexts as a possibly more inclusive approach for older people:

But as a church community, it did an awful lot for us because … and there are a lot of people who cannot come to church because they are disabled anyway, and it opened … it had a benefit in that people said, well if you … we started off by we recorded our services and then we put them on Facebook and our website to start with before we livestreamed and people said, well I can come to church now, which I never could before. (Corinne)

Participants also described how digital devices were used as a type of aide memoire. This included using the diary as a calendar and setting reminders about events and activities that needed to be recalled. The digital also became a memory device when trying to remember some information:

And of course Google … Google is my best friend because I forget things and I can ask Google, she may not always give me the right answer but yeah(!) it gives me an idea. (Katherine)

During the Covid-19 pandemic the amount of digital technologies and devices therefore increased significantly.

it’s been forced on us and we have had to learn to do these things and realized there is a way of communicating when we cannot actually be together. (Pauline)

At a time when movements and mobilities were restricted the use of the digital provided meaningful moments and were mainly viewed as valuable and important.

### 3.2. Social and digital connectivity

Importance of connectivity, that is connections with family, partners, friends, social groups and the locality, as well as wider inter/national communities was expressed by the participants. During the Covid-19 pandemic these connections were predominately maintained virtually with people outside the household due to the public restrictions around movement and social distancing. Many narratives focused on communications and conversations with family, often adult children and grandchildren:

we have got four children and nine grandchildren, so there’s lots of conversations going on all the time when we could not see each other, I mean we had … a grandchild was born during Covid and we did not see him for ages and ages, so it was lovely to be able to Zoom call and Facetime with them. (Corinne)

Some of the participants talked about their caring responsibilities during the Covid-19 pandemic. Digital devices were central to maintaining connectivity for both the participants and the person being cared for:

just as lockdown happened, my mother was in hospital, she’s over 100 now, and she went into … permanently into a care home, and we got her a Facebook Portal, which has got an intelligent camera, so it focuses on the person speaking in the room. And this has been a godsend because also she’s never used any IT whatsoever, she had a very basic mobile phone, and she just can say, hey Portal, call Bob or call Steph, or whatever, and so she can call up any member of the family, any time of day, when she chooses. (Roy)

The sense of time passing during the pandemic was evident from missing being in person around key events within the family, including, the birth of grandchildren, birthdays, Christmas, New Year and seeing grandchildren growing up:

And you know I keep in touch with my daughter, I wasn’t able to see her much, and my grandchildren, and I mean it’s been amazing actually seeing how tall my grandson has got(!) you know from the age of thirteen to fifteen, he’s just shot up and you know I almost do not recognize him when I do manage to see them for a weekend or something. (Katherine)

Whilst the participants were maintaining digital connections, the loss of in-person contact meant that aspects of the social interactions and embodied being-in-the-world were missed by being in different spaces with a screen as a boundary during their only means of communication for some time.

Participants often described ordering their everyday lives to enable enhanced connections with family and friends. This included during their daily routines by planning time to be connected online and also on special occasions, such as, Christmas:

And at Christmas, when we could not meet up, we timed our Christmas dinner to all be at the same time and they set up, I think it was a Teams meeting, anyway, it came through on … I just had to click something and join their meeting and … and then we all had our Christmas dinner together! In our separate houses! Yeah, which was quite nice. (Irene)

Maintaining connections with family, friends and colleagues overseas was however complicated by different time zones that needed to considered:

And one of our members has moved back to the States and so he, not every week, but he joins us, largely because the time difference, when he was working at home it was easier for that but … now he’s back in the office it’s not quite so regular but … that worked very well. (Roy)

Some means of communication were considered convenient and less intrusive as people did not have to be present at the same time. In this context WhatsApp was notably used more, for all types of communication, but also for fun, humour and sharing jokes:

You know and people send … I think that was very uplifting at the beginning, we did send lots of little silly jokes and things and … they are very amusing, you know there’s … especially when there’s a disaster. I do not know why … I do not know how people manage to do it, but you know from some dreadful disaster, somebody makes some sort of joke which you know … I think it releases a tension, does not it. (Katherine)

There were many positive ways that the participants engaged with the digital to enhance their social connections and relationships, at the same time, interspersed through the focus groups, was the sense of loss of human and physical contact with others, often expressed as missing ‘hugs’:

… it was that human contact and hugs that we missed terribly. (Sylvia)

Digital connections have an important purpose but do not replace the sense of being in the same place, at the same time, to have embodied co-presence, and to be in immediate and direct connection with others. The sensate and embodied experiences in everyday life were therefore described as significant:

…there’s just nothing really stands in for face to face contact, I think you pick up far more about, I do not know, just body language and … I know we are speaking and we can hear voice? but it’s … I think it’s just less empathic somehow. (Janet)

In this context, through the narratives, participants also described how the locality and their own neighbourhoods had taken on enhanced meaning and importance through the pandemic. This included using local space more, with or without digital technologies, and meeting more people in the public areas within their locality:

and I discovered lots and lots of places locally that I’d never … I’d no idea they were there, and I mean they take me ten minutes to get there! So that’s been … that’s been really very interesting. (Katherine)

The pandemic also resulted in a period of reflection for many participants. In particular, about the importance of social connections and relations in their everyday lives:

So although it was a really difficult and challenging time and we could not see each other face to face, it did have some … it taught us an awful lot about how we need to care for people and … and what people appreciated and those contacts were so important to the people who were … felt very bereft and on their own. (Corinne)

The disruptive changes from the pandemic resulted in an increased use of the digital in everyday life of the participants. Maintaining social and digital connections was important during this period of limited movements and social distancing. At the same time, the significance of embodied co-presence and the immediacy of shared space and/or time was highlighted.

### 3.3. Challenges and limitations of the digital in everyday life.

There were a number of concerns and limitations around incorporating more and more digital technologies into everyday life. First, not all older people were using digital technologies and participants expressed concerns of some older people feeling excluded. Second, possible issues around scams and privacy were highlighted. Third, participants expressed concerns about the move to more remote connections in health and social care.

Interspersed through the focus groups there were expressions of concerns for older people who were not participating in the online social activities. These interactions discussed how some older people may not feel comfortable with the technology, may be worried about not using digital technologies correctly or did not want or feel able to engage online:

But these people who do have devices but they just will not connect into Zoom, just do not like the technology … But there’s only about five or six of that seventeen who will actually use Zoom socially in that environment, which is so sad. (Shelly)

The resistance expressed by the participants was often within the context of being ‘sad’ as others may be missing out on social activities and connections. In particular, there were some groups within older people who may be at risk of becoming more socially isolated due to limited connections with the digital:

I run a memory café for those with memory issues and dementia and loneliness, which had to stop obviously during Covid and it was quite difficult to … with people with those issues to keep contact. (Corinne)

It may also be that some older people do not have the technologies required or may need assistance in setting up or repairing digital technologies. One participant, for example, explained how for a long time at the start of the pandemic they had technological difficulties that they could not resolve until a younger relative was available:

but what has been really difficult IT wise is that if you do not have … if you do not have anybody handy who can … who you can talk to about your IT issues and things, it … you can really get stuck. And … yeah, have a … have a big problem! …Well the … the battery went on the main mother board in my desktop and … it meant that every time I start … I started it, it had to … you know sort of boot up from scratch and everything. Eventually sorted out when my nephew came on a (laughs) one and only visit and he changed it for me. But I do have IT support, expensive IT support but … had a lot of problem with the printer. (Margaret)

Some participants described how communication on digital devices did not feel natural, and it can be difficult adapting to the momentum and online practices, to ensure good communication:

if there’s more than two of you, it’s a little bit artificial because you are trying to take turns to speak and you worry about talking over people or not participating or whatever, you cannot sort of hug a Zoom image, there’s just nothing really stands in for face to face contact. (Janet)

For others they did not feel confident and knowledgeable about digital and online practices and how to manage these in their daily lives:

My one problem I do have is I’m not very good at you know with sort of e-mails and things like that, but I do not know if that’s what you’d still call a digital world, but getting e-mails and documents and not knowing where to put them. (Katherine)

Many of the participants highlighted their worries about possible scams that seemed to increase as more and more are digitally engaged:

it’s just scam calls mostly, where you can … I’d say ninety nine times out of a hundred, you pick it up, it’s somebody trying to sell you something… well I guess things like the e-mail scams, the text message scams, the ones that ask you to click when you have missed a delivery, there’s a lot of publicity about things like that. (Pauline)

In particular, concerns were expressed about the risk to data and their own privacy with an enhanced sense of vulnerability about who and which communications to trust.

The key concern that the participants highlighted however was the move to more remote means of communications within health and social care during and since the pandemic. This involved less face-to-face appointments with health and social care and instead included aspects of e-health, emails, online calls, texting and emailing, and telephone calls. For some participants the changes had been experienced as effective and efficient:

honestly it was so easy! And he was able to say immediately, I agree with what you said … because I’d already said to him, I think you know this is what it is, and he said, OK fine, he said, just send me a photo, and he agreed, and I was able to go down to the chemist that afternoon and collect my prescription … (Sylvia)

For many participants there was instead a sense of frustration about not being able to connect with health and social care staff in person. Awaiting a zoom or telephone call meant a lot of time waiting just in case, as the timings were often not pre-scheduled. In particular, there was a sense that something might be missed by not being in the same space, at the same time, as the health professionals:

I do not know, it’s just something about being in the same room as the doctor, is not there, that you just … just that feeling that he is, or she is seeing you as actually … you really are, and might pick up things about your condition that they do not see online, that … there’s just that feeling. (Roy)

The importance of embodied co-presence was especially heightened in the context of health and social care staff. This was due to the importance of the sensate and bodily when the participant had queries about their health and wellbeing. If there was a screen mediating online calls, the need to explain symptoms on the telephone and/or to send a photograph online there was a distance and sense of remoteness in the communication and this appeared to result in an increased sense of vulnerability and questions around trust:

whatever you want to have looked at, and I feel like there’s something about … you know like if you look at a photograph of something, you do not always get like the texture of whatever it is you are looking at, whereas in person and … you know especially if you are looking at something that’s raised on your skin or whatever. (Irene)

For many participants there were not only issues around using technologies in the context of intimate and personal health concerns, when the participant was already worried and concerned, but providing information and results remotely, the meaning was not always understood:

but it’s quite difficult to find what you are looking for and to … and also to understand the way they present the results, unless you are a professional, you know, I have to get my daughter to come round and look at it and say, what does this mean, you know?! It’s not very intuitive, it’s not very user-friendly I do not think. (Roy)

Whilst there were many positive experiences and meanings around the increased use of digital technologies in everyday life, there were also exclusions, concerns and vulnerabilities that were experienced and/or observed by the participants. This was especially evident in the use of the digital in the context of health and social care.

## 4. Conclusion

The use of digital technologies and devices increased during and since the pandemic among this sample of older people. The meaning and experiences of digital devices within the narratives are portrayed and contextualized around their own experiences and rhythms of the pandemic. Whilst at the start of the pandemic some participants were initially reluctant about increasing the use of the digital in everyday life, the use, purpose and variety of digital devices increased significantly during and since the Covid-19 pandemic.

Digital devices were viewed as beneficial for maintaining social relationships, social connections, social activities and hobbies, and as a means to organize daily routines as well as an aide memoir. Participants talked about using the digital within wider narratives associated with the pandemic in which daily routines and habits were significantly changed and everyday social contacts outside the household had been lost. The digital was incorporated into their everyday lives as the participants developed new and different rhythms and flows in the context of the wider rhythms of the pandemic (*cf.* Lyon, 2019). Time and space were further interwoven into the narratives and meaning around digital technologies, in which the boundaries around space were continually made and re-made (*cf.* Massey, 1994). In this context the digital provided meaningful moments within the everyday lives of older people and was mainly viewed as valuable and important.

At the same time, digital devices were not viewed as a direct replacement for face-to-face connections and the time during the pandemic highlighted the significance of embodied co-presence. This was notable for older people living alone who lived alone and/or described limited social contacts. Participants also voiced concerns around the risk of scams and privacy and surveillance issues. Of particular note, the changing nature of communications within health and social care was salient, especially the increasing move to remote communications and the loss of face-to-face contacts. In many ways, there was a distinction within the narratives between familiar and localised connections of family and friends and the increasing use of the digital in the health and social care that is expressed in more fearful and vulnerable ways and experienced as depersonalised and disembodied. The nature of the social and embodied connections was therefore meaningful to the participants as well as the space and place in which these occur. As the participants now widen their social contacts post-pandemic, the importance of developing a balance between the use of online and digital means of communication and meeting face-to-face and in person was also highlighted.

Buse (2010) highlighted the ways that narratives of ageing whilst complex often draw upon ideas around the competence of youth and the digital in which there are hierarchies between young and old bodies are reproduced. Within the narratives of the participants there were at times distinctions between young and old around knowledge and expertise of using digital technologies. Discussions around the use of the digital as an aide memoire were often described to mediate a perceived vulnerability around the possibility of being forgetful, rather than a more youthful notion of promoting productivity. The sense of vulnerability and risky old bodies was also evident through the narratives around ageing and the digital. In this context, the narratives reveal how society can naturalise the double standard for the same usage of digital technology between young and old that has possible implications for ageism.

As the research was conducted during the period of the pandemic, when in-person research was restricted, the data was collected online and remotely. There were benefits of this approach and the participants enjoyed the opportunity to reflect on and discuss their experiences online and interactions between participants were engaging. At the same time, other possible participants may have been dissuaded by the remote method of collecting data online. In particular, narratives of older people who did not engage with online social connections and increased their use of digital technologies may be missing. The research presented in this paper can therefore be seen as an account of the experiences and meaning among a diverse group of people, at a certain time and place. The richness of the data has provided important insights into the meaning and experiences of the increasing use of digital devices and technologies during and since the Covid-19 pandemic. Further research that diversifies the sample of older people and includes in-person as well as online data collection would however be fruitful.

## Data availability statement

The raw data supporting the conclusions of this article will be made available by the authors, without undue reservation.

## Ethics statement

The studies involving human participants were reviewed and approved by College of Health, Medicine and Life Sciences Research Ethics Committee, Brunel University London. Reference: 30547-LR-Dec/2021-36960-1. The patients/participants provided their written informed consent to participate in this study.

## Author contributions

All authors listed have made a substantial, direct, and intellectual contribution to the work and approved it for publication.

## References

[ref2] Agrawal S. Dróżdż M. Makuch S. Pietraszek A. Sobieszczańska M. Mazur G. (2021). The assessment of fear of COVID-19 among the elderly population: A cross-sectional study. J. Clin. Med. 10:5537. doi: 10.3390/jcm10235537, PMID: 34884241 PMC8658105

[ref3] Aissaoui N. (2021). The digital divide: a literature review and some directions for future research in light of COVID-19. Glob. Knowl. Mem. Commun. 71, 686–708. doi: 10.1108/GKMC-06-2020-0075

[ref4] Atkinson P. Barker R. (2020). ‘Hey Alexa, what did I forget?’: networked devices, internet search and the delegation of human memory. Convergence 27, 52–65. doi: 10.1177/1354856520925740

[ref1001] Augner C. (2022). Digital divide in elderly: Self-rated computer skills are associated with higher education, better cognitive abilities and increased mental health. The European Journal of Psychiatry, 36, 176–181.

[ref5] Barbour R. (2014) Introducing qualitative research. 2nd edition London: Sage

[ref6] Braun V. Clarke V. (2006). Using thematic analysis in psychology. Qual. Res. Psychol. 3, 77–101. doi: 10.1191/1478088706qp063oa

[ref7] Braun V. Clarke V. (2022) Thematic analysis. A practical guide. London: Sage.

[ref8] Buse C. (2010). E-scaping the ageing body? Computer technologies and embodiment in later life. Ageing Soc. 30, 987–1009. doi: 10.1017/S0144686X10000164

[ref11] Ehni H. J. Wahl H. W. (2020). Six propositions against ageism in the COVID-19 pandemic. J. Aging Soc. Policy 32, 515–525. doi: 10.1080/08959420.2020.1770032 32491963

[ref12] Fraser S. Lagacé M. Bongué B. Ndeye N. Guyot J. Bechard L.. (2020). Ageism and COVID-19: What does our society’s response say about us? Age Ageing 49, 692–695. doi: 10.1093/ageing/afaa097, PMID: 32377666 PMC7239227

[ref13] Freeman S. Marston H. R. Ross C. Morgan D. J. Wilson G. Gates J.. (2022). Progress towards enhanced access and use of technology during the COVID-19 pandemic: A need to be mindful of the continued digital divide for many rural and northern communities. Healthc. Manage. Forum 35, 286–290. doi: 10.1177/08404704221108314 35855623 PMC9301350

[ref15] Gates J. R. Wilson-Menzfeld G. (2022). What role does Geragogy play in the delivery of digital skills programs for middle and older age adults? A systematic narrative review. J. Appl. Gerontol. 41, 1971–1980. doi: 10.1177/07334648221091236 35543169 PMC9364233

[ref17] Giddens A. (1991) Modernity and self identity. Polity Press, Cambridge

[ref18] Goffman E. (1959) The presentation of self in everyday life. Penguin. London

[ref19] Hennink M. Kaiser B. (2022). Sample sizes for saturation in qualitative research: a systematic review of empirical tests. Soc. Sci. Med. 292, 114523–114510. doi: 10.1016/j.socscimed.2021.114523 34785096

[ref21] Kania-Lundholm M. Manchester H. (2022). Ageing with digital technologies: from theory to agency and practice. Int. J. Ageing Later Life 15, 9–21. doi: 10.3384/ijal.1652-8670.4309

[ref22] Katz S. editor (2018) Ageing in everyday life. Materialities and embodiments. Policy Press. Bristol.

[ref23] Kitzinger J. (1994). The methodology of focus groups: the importance of interaction between research participants. Sociol. Health Illn. 16, 103–121. doi: 10.1111/1467-9566.ep11347023

[ref1002] Lager D. Van Hoven B. Huigen P. P. (2016). Rhythms, ageing and neighbourhoods. Environment and Planning A 48, 1565–1580.

[ref24] Lai J. Widmar N. O. (2021). Revisiting the digital divide in the COVID-19 era. Appl. Econ. Perspect. Policy 43, 458–464. doi: 10.1002/aepp.13104, PMID: 33230409 PMC7675734

[ref26] Leder D. (1990) The absent body. Chicago. The University of Chicago Press.

[ref27] Lefebvre H. (2004) Rhythmanalysis. Space, time and everyday life. London: Continuum.

[ref28] Leinonen E. (2022). Everyday life and the new shapes of identities – the different meanings of ‘things that did not happen’ in the lives of Finnish older persons during the pandemic. J. Aging Stud. 62:101052. doi: 10.1016/j.jaging.2022.101052 36008025 PMC9351390

[ref29] Lyon D. (2019) What is rhythmanalysis? Bloomsbury. London

[ref30] Mannheim I. Schwartz E. Xi W. Buttigieg S. C. McDonnell-Naughton M. Wouters E. J.. (2019). Inclusion of older adults in the research and design of digital technology. Int. J. Environ. Res. Public Health 16:3718. doi: 10.3390/ijerph16193718, PMID: 31581632 PMC6801827

[ref31] Mariano J. Marques S. Ramos M. R. Gerardo F. de Vries H. (2020). Too old for computers? The longitudinal relationship between stereotype threat and computer use by older adults. Front. Psychol. 11:568972. doi: 10.3389/fpsyg.2020.568972, PMID: 33123050 PMC7566919

[ref32] Marston H. R. Genoe R. Freeman S. Kulczycki C. Musselwhite C. (2019). Older adults’ perceptions of ICT: Main findings from the technology in later life (TILL) study. Healthcare 7:86. doi: 10.3390/healthcare7030086 31277387 PMC6787574

[ref35] Massey D. (1994) Space, place and gender, Polity Press. Cambridge.

[ref36] McDonough C. C. (2016). The effect of ageism on the digital divide among older adults. J. Gerontol. Geriatr. Med 2, 1–7. doi: 10.24966/GGM-8662/100008

[ref39] Peace S. (2022) The environments of ageing. Space, place and materiality. Bristol: Policy Press.

[ref40] Peine A. Marshall B. L. Martin W. Neven L. (2021) ‘Socio-gerontechnology: interdisciplinary critical studies of ageing and technology’. Routledge. London.

[ref41] Rosales A. Fernández-Ardèvol M. (2020). Ageism in the era of digital platforms. Convergence 26, 1074–1087. doi: 10.1177/1354856520930905, PMID: 33239961 PMC7649948

[ref42] Rossen L. M. Branum A. M. Ahmad F. B. Sutton P. Anderson R. N. (2020). Excess deaths associated with COVID-19, by age and race and ethnicity—United States, January 26–October 3, 2020. Morb. Mortal. Wkly Rep. 69, 1522–1527. doi: 10.15585/mmwr.mm6942e2, PMID: 33090978 PMC7583499

[ref43] Sheng N. Fang Y. Shao Y. Alterman V. Wang M. (2022). The impacts of digital technologies on successful aging in non-work and work domains: an organizing taxonomy. Work Aging Retire 8, 198–207. doi: 10.1093/workar/waac008

[ref44] Shrira A. Hoffman Y. Bodner E. Palgi Y. (2020). COVID-19-related loneliness and psychiatric symptoms among older adults: the buffering role of subjective age. Am. J. Geriatr. Psychiatry 28, 1200–1204. doi: 10.1016/j.jagp.2020.05.018, PMID: 32561276 PMC7251397

[ref45] Swift H. J. Chasteen A. L. (2021). Ageism in the time of COVID-19. Group Process. Intergroup Relat. 24, 246–252. doi: 10.1177/1368430220983452, PMID: 33746563 PMC7941503

[ref46] Taipale S. Oinas T. Karhinen J. (2021). Heterogeneity of traditional and digital media use among older adults: A six-country comparison. Technol. Soc. 66:101642. doi: 10.1016/j.techsoc.2021.101642

[ref47] Turner B. (2004) The new medical sociology: social forms of health and illness WW Norton & Co. New York

[ref48] Twigg J. Martin W. (2015a). The challenge of cultural gerontology. The Gerontologist 55, 353–359. doi: 10.1093/geront/gnu061 24974388

[ref49] Twigg J. Martin W. editors (2015b) Routledge handbook of cultural gerontology. London: Routledge.

[ref50] Van Dijk J. A. (2006). Digital divide research, achievements and shortcomings. Poetics 34, 221–235. doi: 10.1016/j.poetic.2006.05.004

[ref51] Van Dijk J. (2020). The digital divide. Cambridge: John Wiley & Sons.

[ref52] Vargo D. Zhu L. Benwell B. Yan Z. (2021). Digital technology use during COVID-19 pandemic: A rapid review. Hum. Behav. Emerg. Technol. 3, 13–24. doi: 10.1002/hbe2.242

[ref54] Yoo J. Oh J. Kim S. Y. Shin J. Kim S. Roh C. (2022). Impact of digital device, exercise, and music intervention programs on the cognition and depression of the elderly in South Korea: a meta-regression analysis. Int. J. Environ. Res. Public Health 19:4036. doi: 10.3390/ijerph19074036, PMID: 35409720 PMC8998250

